# Ideal cardiovascular health status and preeclampsia: a cohort study

**DOI:** 10.1590/0034-7167-2024-0109

**Published:** 2025-06-20

**Authors:** João Joadson Duarte Teixeira, Bárbara Brandão Lopes, Maria das Graças da Silva Guerreiro, Nádya dos Santos Moura, Lúcia de Fátima da Silva, Rebeca Silveira Rocha, Dafne Paiva Rodrigues, Mônica Oliveira Batista Oriá

**Affiliations:** I Universidade Federal do Ceará. Fortaleza, Ceará, Brazil; II Universidade Estadual do Ceará. Fortaleza, Ceará, Brazil; III Maternidade Escola Assis Chateaubriand. Fortaleza, Ceará, Brazil

**Keywords:** Pre-Eclampsia, Cardiovascular Diseases, Life Style, Disease Prevention, Health Promotion., Preeclampsia, Enfermedades Cardiovasculares, Estilo de Vida: Prevención de Enfermedades, Promoción de la Salud.

## Abstract

**Objectives:**

to evaluate the association between preeclampsia and the cardiovascular health status of pregnant women.

**Methods:**

we conducted a cohort study with 101 pregnant women, following ethical principles and approved by a research ethics committee. We assessed the ideal cardiovascular health using three behaviors (smoking abstinence, body mass index, and physical activity) and three factors (cholesterol, blood pressure, and absence of diabetes and cardiovascular disease). The outcome measured was preeclampsia.

**Results:**

preeclampsia developed in 24.75% of participants, while 9.90% had ideal cardiovascular health. We identified an association between body mass index (p < 0.01) and blood pressure (p < 0.01) with preeclampsia. However, we found no significant association between preeclampsia and overall ideal cardiovascular health status (p > 0.05).

**Conclusions:**

this study highlights the importance of promoting lifestyle changes in this population.

## RESUMEN

**Objetivos:**

verificar la asociación entre el desarrollo de preeclampsia y el estado de salud cardiovascular en mujeres gestantes.

**Métodos:**

estudio de cohorte con 101 gestantes, aprobado por un comité de ética en investigación, cumpliendo con principios éticos. La salud cardiovascular ideal se evaluó mediante tres comportamientos (abstinencia de tabaquismo, índice de masa corporal y actividad física) y tres factores (colesterol, presión arterial y ausencia de diabetes y enfermedad cardiovascular). El desenlace fue la preeclampsia.

**Resultados:**

el 24,75% desarrolló preeclampsia y el 9,90% presentó salud cardiovascular ideal. Se observó una asociación entre índice de masa corporal (p < 0,01) y presión arterial (p < 0,01) con preeclampsia, pero no con el estado de salud cardiovascular ideal (p > 0,05).

**Conclusiones:**

se considera relevante orientar cambios en el estilo de vida de esta población.

## INTRODUCTION

Preeclampsia (PE) is a condition specific to human pregnancy and the most dangerous of the hypertensive disorders of pregnancy (HDP) that occur after the 20th gestational week. It is a leading cause of maternal and perinatal morbidity and mortality worldwide, contributing to over 500,000 fetal deaths and more than 70,000 maternal deaths annually. The prevalence of these disorders ranges from 1% to 14% of pregnancies, with higher rates observed in economically disadvantaged countries^(1,2)^.

For diagnostic purposes, the International Society for the Study of Hypertension in Pregnancy (ISSHP) defines PE as hypertension (systolic blood pressure ≥ 140 mmHg and/or diastolic blood pressure ≥ 90 mmHg) that begins after the 20th week of gestation and coexists with one or more of the following conditions: (1) proteinuria; (2) other maternal organ dysfunctions (renal insufficiency, hepatic involvement, and neurological or hematological complications); or (3) fetal growth restriction due to uteroplacental dysfunction^(3-5)^.

According to the American Heart Association (AHA), key aggravating risk factors associated with cardiovascular risk in women include diabetes mellitus, obesity, and HDP, particularly PE^(6-8)^. For this reason, both the AHA and the Brazilian Society of Cardiology (SBC) recommend primary prevention strategies that emphasize simple cardiovascular health assessments within Primary Health Care, including obstetric history in cardiovascular risk evaluations^(7)^. However, prenatal care providers often underestimate this relationship^(8)^.

In 2010, the American College of Cardiology (ACC) and the AHA introduced a cardiovascular health construct emphasizing behaviors that promote cardiovascular health rather than focusing solely on risk factors that increase the likelihood of developing cardiovascular diseases (CVD). Currently, the AHA offers “Life’s Essential 8”, an updated approach to assessing cardiovascular health that incorporates sleep health as an additional behavior to measure interindividual and intraindividual cardiovascular health^(9)^. Since then, these methods have been encouraged for inclusion in public health policies and community initiatives focusing on primary prevention^(6,9)^.

*Ideal cardiovascular health* is defined by the simultaneous presence of four behaviors and three favorable health factors. The behaviors include (1) abstinence from smoking in the past year, (2) an ideal body mass index (BMI), (3) regular physical activity, and (4) adherence to a dietary pattern that promotes cardiovascular health. The three favorable health factors are (1) untreated total cholesterol ≤ 200 mg/dL, (2) untreated blood pressure ≤ 120/80 mmHg, and (3) the absence of diabetes and clinical CVD^(10)^, emphasizing the primary prevention of these conditions in healthy individuals.

Pregnancy is recognized as an opportune period for assessing the cardiovascular risk associated with PE, aiming to implement health promotion actions focused on primary prevention through lifestyle modifications and adherence to the cardiovascular health standards recommended by the AHA and SBC^(8)^.

Despite international and national public health policies directed at CVD prevention and maternal-child health, as well as global research efforts to understand the relationship between CVD and PE, these conditions remain significant public health challenges worldwide, necessitating substantial investments and further studies for improved understanding. Furthermore, no research to date has specifically explored the association between cardiovascular health and the development of PE.

Considering pregnancy as a window of opportunity, we believe that integrating long-term cardiovascular risk assessment into routine clinical practice, starting in prenatal care, could enable nurses to guide healthy lifestyle counseling and promote activities that encourage ideal cardiovascular health.

## OBJECTIVES

To examine the association between the development of preeclampsia and the cardiovascular health status of pregnant women, seeking evidence for the relationship between CVD risk factors and PE while recognizing pregnancy as an opportunity to identify these risks.

## METHODS

### Ethical considerations

The Research Ethics Committee of the Federal University of Ceará approved the study. We followed ethical and legal principles outlined in Resolutions No. 466/2012 and No. 510/2016 of the Brazilian National Health Council. Participants signed the Informed Consent Form (ICF) or the Assent Form (AF) along with their respective legal guardians when applicable.

### Study design, period, and location

This prospective cohort study followed the guidelines of the Strengthening the Reporting of Observational Studies in Epidemiology (STROBE) statement. Data collection occurred between April 2018 and June 2019 in Fortaleza, the capital of the state of Ceará, Brazil, involving 18 Primary Health Care Units (PHCUs) and 3 public maternity hospitals.

### Population, sample, and inclusion and exclusion criteria

The study population consisted of pregnant women receiving prenatal care at PHCUs. Eligible participants met the following criteria: singleton pregnancy with a gestational age (GA) between 11 weeks and 13 weeks and 6 days, aged 18 or older, or accompanied by a legal guardian if under 18. We excluded women if they had a prior diagnosis of cardiovascular or renal disease, as the concept of ideal cardiovascular health emphasizes primary prevention of these conditions. Women with already established conditions were excluded, as such cases do not align with primary prevention strategies.

This cutoff point was justified as it represents the optimal period for predicting PE using maternal characteristics combined with mean arterial pressure (MAP) and specific biomarkers. This timing enables early identification of high-risk women to initiate prophylactic aspirin therapy before 16 weeks, as recommended by the ASPRE study^(11)^.

We selected the sample based on the inclusion criteria, initially comprising 255 pregnant women. After accounting for losses, exclusions, and dropouts, the final sample consisted of 101 participants.

Dropout occurred under the following conditions: miscarriage; diagnosis of cardiovascular or renal disease during the study; detection of fetal malformations or chromosomal abnormalities identified by researchers; loss of contact with the participant after exhausting all possible means, including three phone call attempts, three WhatsApp message attempts, and checks in the Live Birth Information System (SINASC) and the State Regulation System (UNISUS Web); delivery occurring at facilities not included in the study; and explicit withdrawal by the participant.

### Study protocol

We collected data in two phases during the cohort period from April 2018 to June 2019. A team of trained researchers administered a structured questionnaire designed for this study, as described below.

*Phase 1* - During prenatal care visits at the Primary Health Care Units (PHCUs), we identified eligible pregnant women and administered a 49-item questionnaire, which took an average of five minutes to complete. This phase aimed to gather explanatory variables. BP was measured three times on both arms simultaneously using an internationally validated digital sphygmomanometer (OMRON, model HEM-7113) available in Brazil. BP measurement followed the guidelines of the Seventh Brazilian Guideline for Arterial Hypertension by the Brazilian Society of Cardiology (SBC)^(12)^ and the protocols established by the Fetal Medicine Foundation (FMF), which were the prevailing standards during the study period. Weight (kg) and height (cm) were also measured to calculate BMI using the formula BMI = weight/height^2^ (kg/cm^2^).

We requested that prenatal care professionals order tests assessing cardiovascular health status that are not part of the standard prenatal routine established by Brazil’s Ministry of Health (MH) and the municipal health department. This arrangement was made through agreements with the municipal health department and the involved professionals.

*Phase 2* - Based on the estimated due date (EDD), we contacted participants to confirm the delivery location and obtained obstetric outcome data directly from medical records. The *outcome* was defined as developing any HDP associated with proteinuria, maternal organ dysfunction, or fetal growth dysfunctions indicative of PE. Absence of PE was determined if these conditions were not documented in the patient’s medical records or in the attached copy of the prenatal card.

### Statistical analysis and results interpretation

We performed exploratory and inferential statistical analyses using the Statistical Package for the Social Sciences (SPSS, version 24.0). We adjusted logistic regression models using R software, version 3.5.5.

We considered the following variables for analysis: *Explanatory variables* included age, marital status as per the Brazilian Ministry of Health’s Prenatal Information System (SISPRENATAL), educational attainment, occupation, personal and family income, self-reported race/color, BP, glucose, and cholesterol levels included in the standard prenatal testing routine, and lifestyle-related variables (self-reported regular physical activity prior to pregnancy, BMI adapted for pregnant women with a gestational age up to 14 weeks, as guided by the MH^(13)^, anthropometric measurements taken at the time of participant recruitment, smoking, alcohol use, and other substance use, with these latter variables also self-reported at the time of data collection). The *dependent variable* (outcome) was the presence or absence of any HDP and whether it constituted PE. *HDP* was defined as any BP alteration recorded in more than two measurements.

For diagnostic purposes, this study adhered to the most recent definition of PE provided by the ISSHP in 2014^(3-5)^. *Ideal cardiovascular health* was defined as the simultaneous presence of three favorable health behaviors (abstinence from smoking in the past year, ideal BMI < 25.7 adapted for pregnant women with gestational age up to 14 weeks^(13)^, and regular physical activity) and three favorable health factors (untreated total cholesterol ≤ 200 mg/dL, untreated BP ≤ 120/80 mmHg, and absence of diabetes or glucose < 92 mg/dL)(10). This study did not assess adherence to a dietary pattern promoting cardiovascular or sleep health.

We performed univariate and bivariate analyses as the primary strategy to minimize potential confounding biases. In the bivariate analysis, sociodemographic variables and categorical variables related to the analysis of ideal cardiovascular health status (physical activity, non-smoking, ideal BMI < 25.7 adapted for pregnant women with gestational age up to 14 weeks^(12)^, ideal BP ≤ 120/80 mmHg, glucose, and total cholesterol) were cross-referenced with the development of PE (yes or no). We applied significance tests with a 5% level (p < 0.05) and 95% confidence intervals.

We used the chi-square test to analyze the relationship between PE outcomes and categorical variables. Fisher’s exact test was applied in cases where expected frequencies were less than five. All independent variables with p < 0.20 in bivariate analysis without regression were included in the logistic regression adjustment, retaining only those with p < 0.05. We employed this approach to adjust for potential confounding effects.

We subjected numerical variables to the Kolmogorov-Smirnov test to verify distribution normality and determine the most appropriate measure for use in descriptive and multivariate phases.

## RESULTS

**Figure 1:**
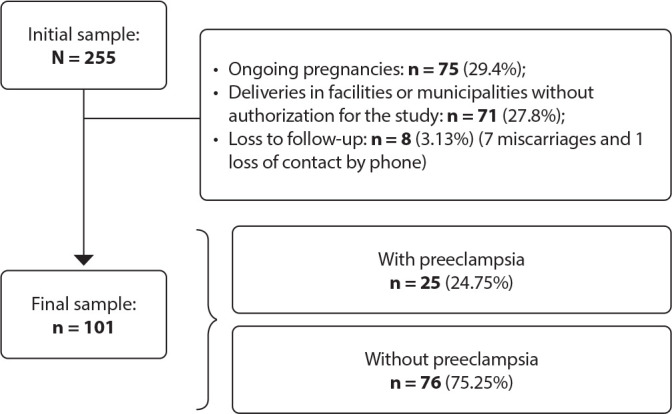
Flowchart of sample selection and proportion of pregnant women who developed preeclampsia (n = 101), Fortaleza, Ceará, Brazil, 2018/2019

As shown in Figure 1, we recruited 255 pregnant women. Among them, 180 gave birth by the conclusion of this study. However, 71 delivered in maternity hospitals or municipalities where study follow-up was not authorized, 7 experienced miscarriage, and 1 was lost to follow-up due to loss of contact, resulting in a final sample of 101 participants. Among the evaluated participants, 24.75% developed PE.

The sample consisted primarily of young women, with a mean age of 24.97 years (±6.35). Most were native to and residing in Fortaleza, Ceará (63.37% and 100%, respectively). The majority identified as homemakers (44.55%), were of mixed race (parda), and had low socioeconomic conditions.

In the bivariate analysis of sociodemographic variables, only household income was associated with the development of PE (p < 0.01). Higher income was correlated with a greater probability of developing PE (Table 1).

**Table 1:** Association between sociodemographic variables and PE (n = 101), Fortaleza, Ceará, Brazil, 2018/2019

Variables	Preeclampsia			*p* value
	Yes (%) (n = 25)	No (%) (n = 76)	Total (%) (n = 101)	
Marital status				
Partner and child(ren)	5 (20.00)	31 (40.79)	36 (35.64)	0.1^^*^^
Partner without child	11 (44.00)	24 (31.58)	35 (34.65)	
Partner, child(ren), and relatives	4 (16.00)	3 (3.95)	7 (6.93)	
Relatives without partner	5 (20.00)	17 (22.37)	22 (21.78)	
Other individuals without marital ties	0 (0.00)	1 (1.32)	1 (0.99)	
Education (in years of schooling)				
1-4	1 (4.00)	2 (2.63)	3 (2.97)	1^^*^^
5-8	4 (16.00)	14 (18.42)	18 (17.82)	
≥ 9	20 (80.00)	60 (78.95)	80 (79.21)	
Personal income (minimum wage)				
No income	10 (40.00)	25 (32.89)	35 (34.65)	0.19^†^
< 1	5 (20.00)	30 (39.47)	35 (34.65)	
1-3	10 (40.00)	21 (27.63)	31 (30.69)	
Family income (minimum wage)		-	-	-
No income	1 (4.00)	2 (2.63)	3 (2.97)	**< 0.01** ^^*^^
< 1	1 (4.00)	15 (19.74)	16 (15.84)	
1-3	16 (64.00)	56 (73.68)	72 (71.29)	
> 3	7 (28.00)	3 (3.95)	10 (9.90)	
Race/Color (self-reported)	-	-	-	-
White	3 (12.00)	11 (14.47)	14 (13.86)	0.52^^*^^
Black	3 (12.00)	3 (3.95)	6 (5.94)	
Mixed	18 (72.00)	58 (76.32)	76 (75.25)	
Asian	1 (4.00)	4 (5.26)	5 (4.95)	
No	0 (0.00)	1 (1.32)	1 (0.99)	

* *Fisher’s exact test; †Chi-square; SM - Brazilian minimum wage in 2018 (R$ 954.00).*

In Table 2, regarding the health-promoting behaviors and cardiovascular health factors, the absence of smoking (n = 92; 91.09%), the presence of ideal BMI (n = 55; 54.45%), ideal blood pressure (n = 83; 82.18%), and ideal total cholesterol levels (n = 39; 38.60%) stand out.

**Table 2:** Cardiovascular health behaviors and factors during the first trimester of pregnancy in cases of PE (n = 101), Fortaleza, Ceará, Brazil, 2018/2019

Variables	Preeclampsia			*p* value
	Yes (%) (n = 25)	No (%) (n = 76)	Total (%) (n = 101)	
Physical activity practice				
Yes	11 (44.00)	32 (42.11)	43 (42.57)	0.87^^*^^
No	14 (56.00)	44 (57.89)	58 (57.43)	
Absence of smoking				
No	1 (4.00)	8 (10.53)	9 (8.91)	0.44^†^
Yes	24 (96.00)	68 (89.47)	92 (91.09)	
Ideal BMI				
Altered (≥ 25.7)	18 (72.00)	28 (36.84)	46 (45.54)	< 0.01^^*^^
Ideal (< 25.7)	7 (28.00)	48 (63.16)	55 (54.45)	
Ideal blood pressure				
Altered	11 (44.00)	7 (9.21)	18 (17.82)	**< 0.01** ^†^
Ideal	14 (56.00)	69 (90.79)	83 (82.18)	
ideal glucose levels				
Altered (≥ 92mg/dL)	2 (8.00)	2 (2.63)	4 (3.96)	0.25^†^
Ideal (< 92 mg/dL)	23 (92.00)	74 (97.37)	97 (96.04)	
Ideal total cholesterol				
Altered	6 (24.00)	17 (22.37)	23 (22.80)	0.97^^*^^
Ideal	10 (40.00)	29 (38.16)	39 (38.60)	
Losses	9 (36.00)	30 (39.47)	39 (38.60)	

* *Chi-square; †Fisher’s exact test; BMI - Body Mass Index.*

As shown in Table 2, when analyzing cardiovascular health factors during the initial phase of the study, ideal BMI (p < 0.01) and ideal blood pressure (p < 0.01) were associated with a lower incidence of PE. However, none of the health-promoting behaviors demonstrated an association with PE development.

In contrast, when cardiovascular health status was cross-referenced with PE outcomes (Table 3), we observed that women with an ideal cardiovascular health status did not develop PE. Of the total participants analyzed, only 10.64% had an ideal or healthy cardiovascular health status, representing 9.90% of the total sample after accounting for the seven exclusions. This analysis showed no significant relationship between ideal cardiovascular health status and PE development (p > 0.05).

**Table 3:** Cardiovascular health status in cases of preeclampsia (n = 101), Fortaleza, Ceará, Brazil, 2018/2019

Variables	Preeclampsia			*p* value
	Yes (%) (n = 23)	No (%) (n = 71)	Total (%) (n = 94^*^)	
Cardiovascular health				
Healthy	0 (0.00)	10 (14.08)	10 (10.64)	0.11†
At least one criterion not met	23 (100.00)	61 (85.92)	84 (89.36)	

* *We considered only cases from the total sample with complete data on cardiovascular health behaviors and factors. Seven cases with missing data for any of the cardiovascular health behaviors or factors were excluded. Tests used: †Fisher’s exact test.*

To better understand the relationships between the analyzed variables, we performed a logistic regression model to estimate the probability of PE occurrence based on these variables (Table 4).

**Table 4:** Results of the logistic model adjustment for predicting preeclampsia development (n = 101), Fortaleza, Ceará, Brazil, 2018/2019

Variables	*Odds Ratio*	95% IC	*p* value
Intercept	0.01	0 -1.46	0.09
Lack of physical activity prior to pregnancy	1.09	0.32-3.73	0.89
BMI	1.20	1.05-1.39	**0.01** ^^*^^
Absence of alcohol consumption	0.19	0.02-1.64	0.12
Altered blood pressure (SBP ≥ 120 mmHg or DBP ≥ 80 mmHg)	13.59	3.26-73.93	**< 0.01** ^^*^^

* *Wald test; BMI - body mass index; SBP - systolic blood pressure; DBP - diastolic blood pressure.*

Only altered BMI and BP were significant in the model (Table 4). We interpreted the significant coefficients using the Odds Ratio. Initially, it is evident that BMI positively affects the likelihood of developing PE, as the Odds Ratio is greater than 1. However, for a more comprehensive interpretation of the parameter estimate, all other variables were held constant and only BMI was varied. By dividing the odds for a one-unit increase in BMI (i.e., BMI value x + 1 divided by BMI value x, where “x” is any fixed value), the result was 1.20. This calculation indicates that with a one-unit increase in BMI, pregnant women have approximately 1.20 times greater odds of receiving a PE diagnosis.

Regarding blood pressure, since the OR is greater than 1, we can infer that normal blood pressure reduces the likelihood of PE. For a clearer interpretation of the relationship between PE diagnosis and the variable “blood pressure”, we divided the odds of PE for pregnant women with altered BP (SBP ≥ 120 mmHg or DBP ≥ 80 mmHg) by the odds for those with ideal BP (SBP < 120 mmHg and DBP < 80 mmHg). This calculation yielded 13.59, indicating that pregnant women with elevated BP were 13.59 times more likely to develop PE than those with ideal BP.

## DISCUSSION

To better understand the context in which this study was conducted, it is relevant to discuss the most significant sociodemographic characteristics of the participating women. Both the 2011 AHA guidelines on the effectiveness of CVD prevention in women and the American College of Obstetricians and Gynecologists (ACOG) guidelines for the prevention of gestational hypertension and PE emphasize the importance of considering disparities related to socioeconomic and cultural characteristics among women as an effective intervention for preventing CVD and PE^(4,7)^.

Given that this study involved pregnant women, the sample was expected to consist of young women, as confirmed by the average age of 24.97 years. The group was predominantly composed of homemakers, women of mixed race, and those with low socioeconomic status. However, the bivariate analysis of sociodemographic variables showed that only family income was associated with PE development, with higher income correlating with a greater likelihood of developing PE. Nevertheless, further studies are recommended to investigate this variable more thoroughly, as lifestyle habits common among individuals with higher family incomes might increase the risk of PE.

Among the sociodemographic characteristics, ACOG identifies African-American race, low socioeconomic status, and maternal age of 35 years or older as risk factors for PE^(4)^. The National Institute for Health and Care Excellence (NICE) considers maternal age of 40 years or older as a risk factor for PE^(5)^, while the ISSHP links certain social determinants of health, such as nutrition and advanced maternal age, to the risk of PE^(3)^.

It is worth noting that international research does not extensively address the influence of marital status or housing conditions on PE. However, a review comparing PE risk factors mentioned in clinical practice guidelines with those from a hierarchical evidence review highlighted the potential association between sociodemographic characteristics and PE development^(14)^. This finding suggests that sociodemographic factors may act as significant stressors and, consequently, as secondary risk factors for both PE and CVD. This is why the AHA recommends investigating and considering these factors in primary CVD prevention and cardiovascular health promotion^(15)^.

The PE incidence in this study was 24.75%, significantly higher than the national and international studies that reported rates ranging from 1% to 14%^(2,15-17)^. However, similar incidence rates were reported in a randomized clinical trial conducted in China, which found 16.8% and 17.1% in the control and intervention groups, respectively, while testing the efficacy of aspirin for preventing PE in high-risk women^(18)^. Another multicenter trial in 13 hospitals across 11 provinces in the study titled “Low-dose aspirin for the prevention of preeclampsia in China (APPEC)” analyzed 889 women with chronic hypertension and reported PE incidences of 25.7% and 27.0% in the control and intervention groups, respectively. However, among women with stage I hypertension, these rates dropped to 16% and 8.2%, and when compared to normotensive women, the rates further decreased to 6.2% and 8.4%, respectively, in the control and intervention groups^(19)^.

The high incidence of PE found in this study could be attributed to the use of updated diagnostic criteria, as recommended by the ISSHP since 2014 and reaffirmed in 2022, which no longer require the presence of proteinuria for a PE diagnosis^(1-3)^. Additionally, the observed rates align with estimates from other studies suggesting that PE incidence in economically disadvantaged areas can reach up to 20%^(20)^.

Regarding cardiovascular health status, the evaluation of this study’s sample can be summarized as follows: 56.44% reported no engagement in physical activity before pregnancy, 91.09% were non-smokers, 82.18% had SBP < 120 mmHg and DBP < 80 mmHg, 96.04% had fasting glucose levels < 92 mg/dL and 38.60% had total cholesterol levels < 200 mg/dL. We collected and measured these data during the first trimester of pregnancy as part of Phase 1 of this study.

The AHA ranks CVD risk factors in order of importance. In descending order, these include smoking, hypertension, high cholesterol, and physical inactivity; subsequently, obesity, diabetes, and poor dietary habits were added^(10)^.

In the bivariate analysis, two variables related to cardiovascular health status were associated with PE development: ideal BMI (p < 0.01) and ideal BP (p < 0.01). Other variables, such as regular physical activity before pregnancy, absence of smoking, and ideal glucose and total cholesterol levels, showed no association with the development of PE in this study’s sample.

None of the women who developed PE had ideal cardiovascular health status, as defined by the AHA’s cardiovascular health behaviors and factors. Only 9.90% of the total sample demonstrated ideal cardiovascular health status, underscoring the need for primary prevention measures to reduce CVD-related morbidity and mortality, as recommended by the AHA^(6)^.

Another population-based study conducted in Brazil with 15,105 active and retired workers aged 35 to 74 years of both sexes observed that less than 1% of the population met the ideal criteria for all cardiovascular health factors studied^(21)^. This finding aligns with the findings of our study. Similarly, recent research conducted in other countries reported comparable results. For example, in Jamaica, researchers conducted a study involving 360 men and 665 women over 20 years old, who participated in a national health and lifestyle survey^(22)^. In Chile, the study included 620 women aged 35 to 70 years without CVD enrolled in a randomized clinical trial testing the efficacy of a mobile short message intervention to improve cardiovascular health^(23)^.

We identified no studies analyzing the relationship between PE development and cardiovascular health status, highlighting the need for more comprehensive research on this topic.

American, European, and Brazilian scientific societies, aiming to meet the Sustainable Development Goals (SDGs) outlined in the 2030 Agenda, have recommended investments in and implementing primary prevention strategies for CVD. This approach is regarded as the most effective and longitudinal prevention method for these diseases. Within this context, the AHA and the ACC advocate for promoting cardiovascular health by managing cardiovascular health metrics, which is believed to contribute to achieving the SDGs related to maternal and infant mortality and deaths from noncommunicable diseases^(4,6,7,10,24,25)^.

In 2011, the AHA formally recognized PE as a clinically significant risk and predictive factor for CVD in women^(7,25)^. Since then, studies have reported the risk association between PE and CVD, emphasizing the importance of controlling risk factors through lifestyle modifications and healthy behaviors, leveraging pregnancy as a window of opportunity for action^(24,26,27)^.

Building on this evidence and recognizing the importance of fostering healthy lifestyles to guide individuals toward ideal cardiovascular health, the ACC and AHA published a 2019 guideline on primary CVD prevention. This guideline focuses on managing lifestyle factors that influence cardiovascular risk and other determinants, emphasizing social determinants of health and optimizing lifestyles to reduce future atherosclerotic CVD events^(6)^.

In the logistic regression model, BMI (p = 0.02; OR: 1.20) and ideal BP < 120/80 mmHg (p < 0.01; OR: 13.59) were confirmed as independent variables for PE development. Recently, American gynecological and obstetric societies have proposed a new BP cutoff point (130/80 mmHg) for diagnosing PE, incorporating AHA recommendations for early detection of the condition^(28)^.

In this context, achieving metrics aligned with ideal cardiovascular health underscores the importance of evaluating preand post-pregnancy CVD risk factors, particularly in women with pregnancies complicated by PE. Health counseling sessions led by prenatal care providers are also essential. As the literature recommends, these sessions should focus on lifestyle modifications and routine cardiovascular evaluations during the postpartum period.

### Study limitations

The main limitation of this study was the lack of access to certain maternity hospitals for outcome data collection. Many women delivered in facilities outside Fortaleza, Ceará, or in private hospitals where data collection was not authorized, influencing the final sample size. Another significant limitation was the inability to perform cholesterol tests, which are not part of standard prenatal care. Consequently, many participants did not complete the test alongside other routine procedures or later.

### Contributions to the field of nursing, health, or public policy

Based on the findings, we suggest further research to reinforce the described data and provide stronger evidence for prenatal care providers, particularly nurses, who play a key role in primary care settings.

Understanding the distribution of preeclampsia cases, the variables associated with this condition, and its impact on cardiovascular health status is crucial for contributing to health and public policy. This knowledge can guide the adoption of lifestyle modifications, beginning with family planning, continuing through prenatal care, and extending into the postpartum period for women who exhibit risk factors for PE or develop the condition during pregnancy. Such measures aim to promote the primary prevention of CVD, thereby reducing cardiovascular risk and improving the cardiovascular health status of this population.

## CONCLUSIONS

In this study, considering the evaluated lifestyle habits, only 9.90% of the pregnant women exhibited ideal cardiovascular health status, while 90.10% presented at least one behavior or risk factor for cardiovascular disease. Among the assessed variables, only elevated BMI and BP exceeding 120/80 mmHg emerged as independent factors for preeclampsia, suggesting an increased cardiovascular risk. We found no association between PE development and cardiovascular health status within the study sample.
