# Supplementary material for: Ideal cardiovascular health status and preeclampsia: a cohort study
Source: Rev Bras Enferm. 2025 Jun 20;78(2):e20240109. doi: 10.1590/0034-7167-2024-0109 (PMC12185144; doi:10.1590/0034-7167-2024-0109)
Supplement: Translation

# Translation: *Status* de saúde cardiovascular ideal e pré-eclâmpsia: um estudo de coorte

**DOI:** 10.1590/0034-7167-2024-0109pt

**Authors:** João Joadson Duarte Teixeira, Bárbara Brandão Lopes, Maria das Graças da Silva Guerreiro, Nádya dos Santos Moura, Lúcia de Fátima da Silva, Rebeca Silveira Rocha, Dafne Paiva Rodrigues, Mônica Oliveira Batista Oriá

**Affiliations:** I Universidade Federal do Ceará. Fortaleza, Ceará, Brasil; II Universidade Estadual do Ceará. Fortaleza, Ceará, Brasil; III Maternidade Escola Assis Chateaubriand. Fortaleza, Ceará, Brasil

**Keywords:** Pré-Eclâmpsia, Doenças Cardiovasculares, Estilo de Vida, Prevenção de Doenças, Promoção da Saúde.

## Abstract

**Objetivos:**

verificar a associação entre desenvolvimento de pré-eclâmpsia e *status* de saúde cardiovascular de gestantes.

**Métodos:**

coorte com 101 gestantes, aprovada em comitê de ética em pesquisa, obedecendo a preceitos éticos. A saúde cardiovascular ideal foi avaliada mediante três comportamentos (abstinência do tabagismo, Índice de Massa Corpórea e atividade física) e três fatores (colesterol, pressão arterial e ausência de diabetes e doença cardiovascular). Desfecho: pré eclâmpsia.

**Resultados:**

24,75% desenvolveram pré-eclâmpsia, e 9,90% tinham saúde cardiovascular ideal. Verificou-se associação entre Índice de Massa Corpórea (p < 0,01) e pressão arterial (p < 0,01) com pré-eclâmpsia, mas não com *status* de saúde cardiovascular ideal (p > 0,05).

**Conclusões:**

considera-se relevante orientar mudanças no estilo de vida dessa população.

## INTRODUÇÃO

A pré-eclâmpsia (PE) é uma doença específica da gestação humana e o mais perigoso dos distúrbios hipertensivos gestacionais (DHG) que ocorrem após a 20ª semana gestacional. É responsável por altas taxas de morbimortalidade materna e perinatal em todo o mundo, ocasionando mais de 500 mil mortes fetais e mais de 70 mil mortes maternas por ano. A prevalência desses distúrbios pode variar entre 1% e 14% das gestantes, atingindo patamares mais altos nos países menos favorecidos economicamente^(1,2)^.

Para fins diagnósticos, a *International Society for the Study of Hypertension in Pregnancy* (ISSHP) define a PE como sendo a presença de hipertensão (pressão arterial sistólica - PAS ≥ 140 mmhg e/ou pressão arterial diastólica - PAD ≥ 90 mmHg) iniciada após a 20ª semana de idade gestacional (IG) e coexistência de uma ou mais das seguintes condições: 1. Proteinúria; 2. Outras disfunções orgânicas maternas (insuficiência renal, envolvimento hepático, complicações neurológicas e complicações hematológicas); ou 3. Disfunção do crescimento fetal por disfunção uteroplacentária^(3-5)^.

Segundo a American Heart Association (AHA), os principais fatores de risco agravantes para associação ao risco cardiovascular na população feminina são a ocorrência de diabetes *mellitus* ou de obesidade ou dos DHG, com destaque para a PE^(6-8)^. Por isso, a AHA e a Sociedade Brasileira de Cardiologia (SBC) recomendam a prevenção primária baseada na abordagem de fácil execução da saúde cardiovascular no âmbito da Atenção Primária em Saúde, incluindo a história obstétrica na avaliação de risco cardiovascular^(7)^. Porém, os agentes promotores de cuidados pré-natais ainda subestimam muito essa relação^(8)^.

Em 2010, o American College of Cardiology (ACC) e a AHA definiram um constructo de Saúde Cardiovascular focado em comportamentos que promovem a saúde cardiovascular em vez de fatores de risco que aumentam a probabilidade de desenvolver doenças cardiovasculares (DCV). Atualmente a AHA disponibiliza o *Life’s Essential 8*, que apresenta uma abordagem aprimorada para avaliar a saúde cardiovascular, acrescentando a saúde do sono como comportamento para medir a saúde cardiovascular interindividual e intraindividual^(9)^. Desde então, esses métodos para medir a saúde cardiovascular vêm sendo estimulados a serem incorporados às políticas de saúde pública e comunidade, com foco na prevenção primária^(6,9)^.

Para tanto, define-se por Saúde Cardiovascular Ideal a presença simultânea de quatro comportamentos e três fatores favoráveis à saúde. Os comportamentos são: 1. Abstinência do tabagismo no último ano; 2. Índice de Massa Corpórea (IMC) ideal; 3. Prática regular de atividade física; e 4. Consumo de um padrão alimentar que promova a saúde cardiovascular. Já os três fatores favoráveis à saúde são: 1. Colesterol total não tratado ≤ 200 mg/dL; 2. Pressão arterial (PA) não tratada ≤ 120/80 mmHg; e 3. Ausência de diabetes e de DCV clínica^(10)^ - por envolver a prevenção primária do desenvolvimento dessas patologias em pessoas saudáveis.

A gestação é reconhecida como um momento conveniente para a investigação do risco cardiovascular associado à PE, visando implementar ações de promoção da saúde voltadas à prevenção primária mediante incentivo de mudanças no estilo de vida e adequação ao *status* de saúde cardiovascular recomendado pela AHA e pela SBC^(8)^.

Apesar das políticas públicas internacionais e nacionais de saúde direcionadas à prevenção das DCV e à saúde materno-infantil, além das pesquisas desenvolvidas globalmente buscando entender a relação entre as DCV e PE, essas doenças ainda são importantes problemas de saúde pública em todo o mundo, requerendo muitos investimentos e mais estudos para melhor entendê-las. Como acréscimo, vale dizer que desconhecemos pesquisas associando a saúde cardiovascular ao desenvolvimento de PE.

Assim, considerando a gestação como uma janela de oportunidades, acreditamos que a incorporação da avaliação de risco cardiovascular de longo prazo pelo enfermeiro na prática clínica de rotina desde o pré-natal pode orientar o aconselhamento do estilo de vida saudável, visando a atividades promotoras da Saúde Cardiovascular Ideal.

## OBJETIVOS

Verificar a associação entre desenvolvimento de pré-eclâmpsia e *status* de saúde cardiovascular de gestantes, com a busca de evidências da associação entre os fatores de risco para DCV e PE, reconhecendo a gravidez como oportunidade para identificá-los.

## MÉTODOS

### Aspectos éticos

A pesquisa foi aprovada pelo Comitê de Ética em Pesquisa da Universidade Federal do Ceará. Foram respeitados os princípios éticos e legais preconizados pelas Resoluções nº 466/2012 e nº 510/2016 do Conselho Nacional de Saúde. As participantes da pesquisa assinaram o Termo de Consentimento Livre e Esclarecido (TCLE) ou o Termo de Assentimento Livre e Esclarecido (TALE) juntamente com seus respectivos responsáveis.

### Desenho, período e local do estudo

Trata-se de um estudo observacional de coorte prospectiva norteado pelo *STrengthening the Reporting of OBservational studies in Epidemiology* (STROBE). A coorte se deu entre abril de 2018 e junho de 2019, no município de Fortaleza, estado do Ceará (CE), Brasil, envolvendo 18 Unidades de Atenção Primária à Saúde (UAPS) e 3 maternidades públicas.

### População e Amostra, critérios de inclusão e exclusão

A população foi constituída de gestantes que faziam pré-natal nas UAPS, sendo elegíveis aquelas com as seguintes características: ter gestação única com IG entre 11 semanas e 13 semanas e 6 dias, além de ter 18 anos ou estar acompanhada de seu responsável legal se tivesse menos de 18 anos. Foram tidas como inelegíveis: gestantes com doença cardiovascular ou renal diagnosticadas previamente à pesquisa, já que a abordagem da Saúde Cardiovascular Ideal visa a uma estratégia de prevenção primária desses agravos; e pessoas com esses agravos já estabelecidos, pois tais casos fogem à prevenção primária.

Esse ponto de corte da IG justifica-se por ser o melhor período para implementar a predição da PE por características maternas associada à pressão arterial média (PAM) e biomarcadores específicos em tal período gestacional. O intuito é identificar as mulheres com maior risco, a fim de iniciar terapia profilática com ácido acetilsalicílico (AAS) em tempo hábil, antes de 16 semanas, conforme recomendado pelo estudo ASPRE^(11)^.

A amostra foi selecionada com base nos critérios de inclusão acima descritos, sendo captadas 255 gestantes inicialmente. Após as perdas, aplicação dos critérios de exclusão e descontinuidade, a amostra final foi de 101 pacientes.

Foram apontados como critérios de descontinuidade: aborto; gestantes com DCV ou doença renal diagnosticada durante a pesquisa; más-formações fetais ou anormalidades cromossômicas conhecidas dos pesquisadores em algum momento da pesquisa; perda de contato com a gestante após esgotadas todas as possibilidades de contato, sendo três tentativas de contato telefônico, três tentativas de contato por mensagem via WhatsApp e verificação no Sistema de Informação de Nascidos Vivos (SINASC) e no Sistema Estadual de Regulação (UNISUS Web); ocorrência do parto em unidades diferentes daquelas envolvidas no estudo; e desistência expressa pela participante.

### Protocolo do estudo

A coleta de dados foi realizada no período da coorte, de abril de 2018 a junho de 2019, em dois momentos. Uma equipe de pesquisadores devidamente treinada aplicou um formulário estruturado e elaborado especificamente para o estudo, conforme descrição a seguir.

*Momento 1* - Durante a assistência pré-natal nas UAPS, foram identificadas as gestantes elegíveis e aplicado o formulário de 49 itens em um tempo médio de cinco minutos, buscando as variáveis explanatórias. A PA foi verificada três vezes em ambos os braços simultaneamente, com esfigmomanômetro digital da marca OMRON e modelo HEM-7113, validado internacionalmente e disponível no Brasil. A técnica de aferição foi aplicada de acordo com as orientações da VII Diretriz Brasileira de Hipertensão Arterial da Sociedade Brasileira de Cardiologia (SBC)^(12)^ e protocolos instituídos pela Fetal Medicine Foundation (FMF), diretrizes vigentes na ocasião da pesquisa. Também foram mensurados peso (kg) e altura (cm) para cálculo do IMC seguindo a fórmula *IMC = peso / altura2 (kg/cm2)*. Foram solicitados aos profissionais pré natalistas os exames para avaliação do *status* de saúde cardiovascular que não fazem parte da rotina de pré-natal estabelecida pelo Ministério da Saúde (MS) do Brasil e pela Secretaria da Saúde do Município, conforme acordo firmado com a secretaria municipal de saúde e com os respectivos profissionais.

*Momento 2* - De acordo com a data provável do parto (DPP), entrou-se em contato com as participantes para conhecimento do local e busca de dados sobre o desfecho obstétrico diretamente nos prontuários. Foi considerado “desfecho” o desenvolvimento de algum DHG associado à proteinúria ou disfunções orgânicas maternas ou, ainda, disfunções do crescimento fetal que possibilitassem diagnosticar PE. Foi considerado ausência de PE quando esses eventos não estavam registrados no prontuário da paciente nem na cópia do cartão de pré-natal anexada ao prontuário.

### Análise dos resultados e estatística

Nas fases exploratória e inferencial, as análises estatísticas dos dados foram realizadas com o auxílio do *software Statistical Package for the Social Sciences* (SPSS, versão 24.0). A exceção vai para o modelo de regressão logístico, que foi ajustado com uso do software R, versão 3.5.5.

Para fins de análise, foram consideradas as seguintes variáveis: *Explanatórias* - idade, estado conjugal de acordo com o Sistema de Informação de Pré-natal (SISPRENATAL) do MS do Brasil, escolaridade, ocupação, renda pessoal e familiar, raça/cor autorreferida, PA, glicemia e colesterol incluídos na rotina de exames do pré-natal e as variáveis relativas ao estilo de vida (qualquer prática regular de atividade física referida no período pré-gestacional, IMC adaptado para gestantes com IG até 14 semanas, como orientado pelo MS^(13)^, cujos dados antropométricos foram mensurados no momento da captação das gestantes, bem como tabagismo e uso de álcool e outras drogas, sendo essas últimas variáveis também autorreferidas no momento da coleta de dados); e *Variável Dependente (Desfecho)* - presença ou não de algum DHG e se este seria ou não uma PE, sendo considerado DHG qualquer alteração de PA em mais de duas aferições.

Para fins diagnósticos de PE, foi considerada a definição mais atual dada pela *International Society for the Study of Hypertension in Pregnancy* (ISSHP) em 2014^(3-5)^. O estudo considerou “Saúde Cardiovascular Ideal” a presença simultânea de três comportamentos favoráveis à saúde (1. Abstinência do tabagismo no último ano; 2. Índice de Massa Corpórea ou IMC Ideal < 25,7 - adaptado para gestantes com IG até 14 semanas^(13)^; e 3. Prática regular de atividade física) e de três fatores favoráveis à saúde (1. Colesterol total não tratado ≤ 200 mg/dL; 2. Pressão arterial não tratada ≤ 120/80 mmHg; e 3. Ausência de diabetes ou glicemia < 92 mg/dL)^(10)^. Para este estudo, não foi avaliado o consumo de um padrão alimentar que promova a saúde cardiovascular nem a saúde do sono.

Foram desenvolvidas as análises univariada e bivariada, principal estratégia utilizada para a minimização dos possíveis vieses de confusão. Na análise bivariada, as variáveis sociodemográficas e as variáveis categóricas relacionadas à análise do *status* de saúde cardiovascular ideal [prática de atividade física, ausência de tabagismo, IMC Ideal < 25,7 - adaptado para gestantes com IG até 14 semanas^(12)^, PA ideal (≤ 120/80 mmHg), glicemia e colesterol total ideal], foram cruzadas com o desenvolvimento de PE (sim ou não). Foram aplicados testes de significância com nível de 5% (p < 0,05) e intervalo de confiança de 95%.

Aplicou-se o teste qui-quadrado para os cruzamentos do desfecho de PE com variáveis de natureza categórica. Foi empregado o teste exato de Fisher nos casos em que as frequências esperadas foram inferiores a 5. Todas as variáveis independentes que apresentaram *p* < 0,20 na análise bivariada sem regressão foram inseridas no ajuste de regressão logístico, permanecendo nele aquelas que alcançaram *p* < 0,05. Essa estratégia foi utilizada para ajustar os possíveis efeitos de confusão.

As variáveis numéricas foram submetidas ao teste de Kolmogorov-Smirnov para verificar a normalidade de distribuição e determinar a melhor medida a ser utilizada, seja na fase descritiva, seja na multivariada.

## RESULTADOS

Conforme demonstrado na Figura 1, foram captadas 255 gestantes. Entre elas, 180 pariram até a conclusão deste estudo, sendo que 71 pariram em maternidades ou municípios onde não havia autorização para seguimento do estudo, 7 tiveram aborto e 1 não foi localizada (perda de contato), restando a amostra final de 101 pacientes. Entre as gestantes avaliadas, 24,75% tiveram PE.

**Figura 1:** Fluxograma de obtenção da amostra e proporção de gestantes que desenvolveram pré eclâmpsia (n = 101), Fortaleza, Ceará, Brasil, 2018/2019

A amostra caracterizou-se por ser de mulheres jovens, com média de idade de 24,97 anos (±6,35) e naturais (63,37%) e residentes (100%) de Fortaleza - CE. A maioria tinha como principal ocupação ser dona de casa (44,55%), era parda e apresentava baixas condições socioeconômicas.

Entre as variáveis sociodemográficas, a análise bivariada revelou que apenas a renda familiar esteve associada ao desenvolvimento de PE (p < 0,01), de modo que a maior renda associou-se à maior probabilidade de desenvolvimento da PE (Tabela 1).

**Tabela 1:** Associação entre as variáveis sociodemográficas e a pré-eclâmpsia (n = 101), Fortaleza, Ceará, Brasil, 2018/2019

Variáveis	Pré-eclâmpsia			Valor de *p*
	Sim (%) (n = 25)	Não (%) (n = 76)	Total (%) (n = 101)	
Estado conjugal				
Companheiro e filho(s)	5 (20,00)	31 (40,79)	36 (35,64)	0,1^^*^^
Companheiro sem filho	11 (44,00)	24 (31,58)	35 (34,65)	
Companheiro, filhos e familiares	4 (16,00)	3 (3,95)	7 (6,93)	
Familiares sem companheiro	5 (20,00)	17 (22,37)	22 (21,78)	
Outras pessoas sem laços conjugais	0 (0,00)	1 (1,32)	1 (0,99)	
Escolaridade (em anos de estudo)				
1-4	1 (4,00)	2 (2,63)	3 (2,97)	1^^*^^
5-8	4 (16,00)	14 (18,42)	18 (17,82)	
≥ 9	20 (80,00)	60 (78,95)	80 (79,21)	
Renda pessoal (SM)				
Sem renda	10 (40,00)	25 (32,89)	35 (34,65)	0,19^†^
< 1	5 (20,00)	30 (39,47)	35 (34,65)	
1-3	10 (40,00)	21 (27,63)	31 (30,69)	
Renda familiar (SM)		-	-	-
Sem renda	1 (4,00)	2 (2,63)	3 (2,97)	**< 0,01** ^^*^^
< 1	1 (4,00)	15 (19,74)	16 (15,84)	
1-3	16 (64,00)	56 (73,68)	72 (71,29)	
> 3	7 (28,00)	3 (3,95)	10 (9,90)	
Raça/Cor (autorreferida)	-	-	-	-
Branca	3 (12,00)	11 (14,47)	14 (13,86)	0,52^^*^^
Negra	3 (12,00)	3 (3,95)	6 (5,94)	
Parda	18 (72,00)	58 (76,32)	76 (75,25)	
Amarela	1 (4,00)	4 (5,26)	5 (4,95)	
Não	0 (0,00)	1 (1,32)	1 (0,99)	

^^*^^Exato de Fisher;

† Qui-quadrado; SM - salário mínimo brasileiro em 2018 (R$ 954,00).

Na Tabela 2, no tocante aos comportamentos e fatores promotores da saúde cardiovascular, destaca se a ausência de tabagismo (n = 92; 91,09%), a presença de IMC ideal (n = 55; 54,45%), os níveis pressóricos ideais (n = 83; 82,18%) e o colesterol total ideal (n = 39; 38,60%).

**Tabela 2:** Comportamentos e fatores de saúde cardiovascular no primeiro trimestre gestacional em casos de pré-eclâmpsia (n = 101), Fortaleza, Ceará, Brasil, 2018/2019

Variáveis	Pré-eclâmpsia			Valor de *p*
	Sim (%) (n = 25)	Não (%) (n = 76)	Total (%) (n = 101)	
Prática de atividade física				
Sim	11 (44,00)	32 (42,11)	43 (42,57)	0,87^^*^^
Não	14 (56,00)	44 (57,89)	58 (57,43)	
Ausência de tabagismo				
Não	1 (4,00)	8 (10,53)	9 (8,91)	0,44^†^
Sim	24 (96,00)	68 (89,47)	92 (91,09)	
IMC ideal				
Alterado (≥ 25,7)	18 (72,00)	28 (36,84)	46 (45,54)	< 0,01^^*^^
Ideal (< 25,7)	7 (28,00)	48 (63,16)	55 (54,45)	
Pressão arterial ideal				
Alterada	11 (44,00)	7 (9,21)	18 (17,82)	**< 0,01** ^†^
Ideal	14 (56,00)	69 (90,79)	83 (82,18)	
Glicemia ideal				
Alterada (≥ 92mg/dL)	2 (8,00)	2 (2,63)	4 (3,96)	0,25^†^
Ideal (< 92 mg/dL)	23 (92,00)	74 (97,37)	97 (96,04)	
Colesterol total ideal				
Alterado	6 (24,00)	17 (22,37)	23 (22,80)	0,97^^*^^
Ideal	10 (40,00)	29 (38,16)	39 (38,60)	
Perdas	9 (36,00)	30 (39,47)	39 (38,60)	

^^*^^Qui-quadrado;

† Exato de Fisher; IMC - Índice de Massa Corpórea.

Ainda de acordo com a Tabela 2, na análise dos fatores de saúde cardiovascular no primeiro momento da pesquisa, foi observada a associação das variáveis IMC ideal (p < 0,01) e PA ideal (p < 0,01) com o menor desenvolvimento de PE, ao passo que nenhum dos comportamentos promotores de saúde cardiovascular mostrou associação com o desenvolvimento de PE.

No entanto, quando cruzado o *status* de saúde cardiovascular com o resultado da PE (Tabela 3), observou-se que todas as mulheres com um *status* de saúde cardiovascular saudável não tiveram PE. Do total analisado, apenas 10,64% tinham *status* de saúde cardiovascular ideal ou saudável, o que representou 9,90% da amostra total, tendo em vista que houve sete perdas. Nessa análise, não houve relação entre o *status* de saúde cardiovascular ideal e o desenvolvimento de PE (p > 0,05).

**Tabela 3:** *Status* de saúde cardiovascular nos casos de pré-eclâmpsia (n = 101), Fortaleza, Ceará, Brasil, 2018/2019

Variáveis	Pré-eclâmpsia			Valor de *p*
	Sim (%) (n = 23)	Não (%) (n = 71)	Total (%) (n = 94^*^)	
Saúde cardiovascular				
Saudável	0 (0,00)	10 (14,08)	10 (10,64)	0,11†
Pelo menos um critério não atingido	23 (100,00)	61 (85,92)	84 (89,36)	

* *Foram considerados apenas os casos da amostra total que tinham todos os dados relativos aos comportamentos e fatores de saúde cardiovascular. Assim, foram excluídos sete casos com dados faltantes para algum dos critérios relativos aos comportamentos e fatores de saúde cardiovascular. Testes utilizados: †Exato de Fisher.*

Para entender melhor as relações existentes entre as variáveis em análise, foi realizado um modelo de regressão logística com o objetivo de modelar a probabilidade de ocorrência da PE em função dessas variáveis (Tabela 4).

**Tabela 4:** Resultados do ajuste do modelo logístico para modelar o desenvolvimento de pré eclâmpsia (n = 101), Fortaleza, Ceará, Brasil, 2018/2019

Variáveis	*Odds Ratio*	IC 95%	Valor de *p*
Intercepto	0,01	0 -1,46	0,09
Ausência de atividade física antes da gravidez	1,09	0,32-3,73	0,89
IMC	1,20	1,05-1,39	**0,01** ^^*^^
Ausência de alcoolismo	0,19	0,02-1,64	0,12
Pressão arterial alterada (PAS ≥ 120 mmHg ou PAD ≥ 80 mmHg)	13,59	3,26-73,93	**< 0,01** ^^*^^

* *Teste de Wald; IMC - Índice de Massa Corpórea; PAS - pressão arterial sistólica; PAD - pressão arterial diastólica.*

Apenas o IMC e a PA alterados mostraram-se significantes no modelo (Tabela 4). Então, foram realizadas as interpretações dos coeficientes significantes com uso de *Odds Ratio*. De início, já se sabe que o impacto do IMC na probabilidade de desenvolvimento de PE é positivo, pois a *Odds Ratio* é maior que 1. Porém, para uma interpretação mais ampla da estimativa do parâmetro, as demais variáveis foram fixadas, e apenas o IMC foi variado. Então, dividiram-se as chances caso a gestante tivesse a variação de 1 ponto de IMC, isto é, valor de IMC x + 1 dividido por IMC x (x é um valor fixo qualquer, apenas para interpretação), que resultou em 1,20. Isso quer dizer que, com o acréscimo de 1 ponto no valor de IMC, as gestantes apresentam aproximadamente 1,20 vez mais chance de ter diagnóstico de PE.

Já em relação à PA, como a *Odds Ratio* é maior que 1, pode-se dizer que a PA normal diminui as chances de PE. Para melhor interpretação da relação do diagnóstico de PE com a variável “pressão arterial”, dividiram-se as chances, caso a gestante tivesse tido PA alterada (PAS ≥ 120mmHg ou PAD ≥ 80mmHg), pela chance de a gestante ter tido PA ideal (PAS < 120 mmHg ou PAD < 80 mmHg), resultando em 13,59. Esse resultado indica que as gestantes com PA alta tiveram 13,59 vezes mais chances de desenvolver PE do que as gestantes que tinham PA ideal.

## DISCUSSÃO

Para entender melhor em que contexto o estudo foi realizado, é pertinente discutir as características sociodemográficas mais importantes das mulheres envolvidas nele. Tanto as diretrizes da AHA (atualização 2011) baseadas na eficácia para a prevenção de DCV em mulheres quanto as do *American College of Obstetricians and Gynecologists* (ACOG) relacionadas à prevenção da hipertensão gestacional e da PE reconhecem a importância de observar as disparidades relacionadas às características socioeconômicas e culturais na população feminina como uma intervenção efetiva na prevenção das DCV e da PE^(4,7)^.

Assim, por se tratar de um estudo envolvendo gestantes, era previsível um grupo de mulheres jovens, confirmado pela média de idade de 24,97 anos. Tal grupo foi composto por donas de casa, pardas e com baixa situação socioeconômica. No entanto, de acordo com a análise bivariada das variáveis sociodemográficas, apenas a renda familiar esteve associada com o desenvolvimento de PE, de modo que a maior renda associou-se à maior probabilidade de desenvolver a PE. Porém, recomenda-se que outros estudos avaliem melhor essa variável, já que outros fatores, como hábitos de vida próprios das pessoas com essa renda familiar, podem aumentar o risco para PE.

Dentre as características sociodemográficas, o ACOG relaciona, entre os fatores de risco para PE, a raça afro-americana, o baixo *status* socioeconômico e a idade materna de 35 anos de idade ou mais^(4)^. O *National Institute for Health and Care Excellence* (NICE) reconhece como fator de risco para PE a idade materna de 40 anos ou mais^(5)^, enquanto a ISSHP relaciona como fatores de risco para PE alguns determinantes sociais da saúde, como nutrição e idade materna avançada^(3)^.

Vale salientar que pesquisas internacionais não abordam muito a influência da situação conjugal e das condições de moradia da mulher sobre a PE. Todavia, um estudo de revisão que comparou os fatores de risco da PE referidos pelas diretrizes de prática clínica com os fatores de risco de uma revisão de evidências hierárquicas mostrou provável a possível associação das características sociodemográficas com o desenvolvimento de PE^(14)^. Acredita-se que esta possa ser uma importante variável causadora de estresse e, consequentemente, um fator de risco secundário, tanto para PE como para DCV, tanto que é recomendado pela AHA essa investigação e ponderação quando da prevenção primária de DCV e da promoção da saúde cardiovascular^(15)^.

A incidência de PE encontrada foi de 24,75%, um percentual bem acima dos estudos nacionais e internacionais, que indicam variação de 1% a 14%^(2,15-17)^. No entanto, o que mais se aproxima dessas taxas de incidência foi a incidência de 16,8% e 17,1%, respectivamente encontradas em grupo-controle e em grupo-intervenção de um ensaio clínico randomizado realizado na China para testar a eficácia do ácido acetilsalicílico na prevenção de PE em mulheres de alto risco^(18)^. Outro ensaio multicêntrico em 13 hospitais de 11 províncias no estudo “Ácido acetilsalicílico em baixas doses na prevenção da PE na China (APPEC)”, utilizando uma coorte de 889 mulheres hipertensas crônicas, encontrou taxas de incidências de 25,7% e 27,0%, respectivamente, no grupo-controle e no grupo-intervenção. Porém, quando avaliaram apenas mulheres com hipertensão estágio I, tais taxas caíram para 16% e 8,2%; e quando comparadas com mulheres normotensas, houve uma redução ainda maior para 6,2% e 8,4%, respectivamente no grupo-controle e no grupo-intervenção^(19)^.

A alta incidência de PE encontrada neste estudo pode dever-se ao fato de terem sido considerados novos critérios diagnósticos, conforme orientado pela ISSHP desde 2014 e reafirmados em 2022, segundo os quais não é mais necessário presença de proteinúria para fechar diagnóstico de PE^(1-3)^. Além disso, os valores encontrados aproximam-se das estimativas de outro estudo segundo o qual em lugares menos favorecidos essa incidência pode chegar a 20%^(20)^.

Quanto ao *status* de saúde cardiovascular, a avaliação da presente amostra pode ser caracterizada da seguinte forma: não referiam realização de atividade física no período pré gestacional (56,44%), não fumavam (91,09%), tinham PAS < 120mmHg e PAD < 80mmHg (82,18%), tinham níveis de glicemia em jejum < 92mg/dL (96,04%) e colesterol total < 200mg/dL (38,60%). Tais dados foram coletados e aferidos no primeiro trimestre gestacional por ocasião do Momento 1 da pesquisa.

A AHA considera os fatores de risco para DCV segundo uma ordem de importância. Em ordem decrescente, estão o tabagismo, hipertensão arterial, colesterol alto e inatividade física; em seguida, foram adicionados obesidade, diabetes e hábitos dietéticos ruins^(10)^.

Na análise bivariada, observou-se a associação de duas variáveis de análise do *status* de saúde cardiovascular com o desenvolvimento de PE: IMC ideal (p < 0,01) e PA ideal (p < 0,01). As demais variáveis, como prática regular de atividade física no período pré-gestacional, ausência de tabagismo, níveis ideais de glicemia e colesterol total, não tiveram qualquer tipo de associação com o desenvolvimento de PE na amostra deste estudo.

Nenhuma das mulheres que apresentou PE tinha o *status* de saúde cardiovascular ideal baseado nos comportamentos e fatores de saúde cardiovascular propostos pela AHA e no estilo de vida saudável. Apenas 9,90% da amostra total tinham *status* de saúde cardiovascular ideal, evidenciando, assim, a necessidade de atuar na prevenção primária para diminuir a morbimortalidade por DCV, conforme orientado pela AHA^(6)^.

Outra pesquisa de base populacional realizada no Brasil com 15.105 trabalhadores ativos e aposentados, entre 35 e 74 anos, de ambos os sexos, observou que menos de 1% da população apresentou cenário ideal em todos os fatores estudados para saúde cardiovascular^(21)^, o que se alinha, portanto, com os dados aqui encontrados. O achado da pesquisa citada converge também com o de estudos realizados mais recentemente em outros países. Por exemplo, na Jamaica, pesquisadores conduziram um estudo com 360 homens e 665 mulheres, maiores de 20 anos, participantes de um inquérito nacional relativo à saúde e estilo de vida^(22)^. Já no Chile, a pesquisa contou com 620 mulheres de 35 a 70 anos, sem DCV, inscritas em um ensaio clínico randomizado para testar a eficácia de uma intervenção por mensagens curtas de celular visando à melhoria da saúde cardiovascular^(23)^.

Nenhuma pesquisa foi encontrada analisando o desenvolvimento de PE com o *status* de saúde cardiovascular, evidenciando a necessidade de se investir em pesquisas com um maior alcance e com esse objetivo.

Sociedades científicas americanas, europeias e brasileiras, no intuito de alcançar as metas dos Objetivos do Desenvolvimento Sustentável (ODS) propostos na Agenda 2030, têm recomendado investimentos e implementação de ações na prevenção primária de DCV, que é vista como a prevenção mais efetiva e longitudinal dessas doenças. É nesse contexto que a AHA e o ACC sugerem a promoção da saúde cardiovascular mediante o controle das medidas de saúde cardiovascular, o que se acredita contribuir para o alcance das metas dos ODS relativas à mortalidade materno-infantil e às mortes por doenças não transmissíveis^(4,6,7,10,24,25)^.

Em 2011, a AHA passou a reconhecer formalmente a PE como risco clinicamente importante e fator preditivo de DCV em mulheres^(7,25)^. Desde então, pesquisas têm relatado a associação de risco entre PE e DCV e reconhecido a importância do controle dos fatores de risco por meio de recomendações de adequações do estilo de vida a comportamentos saudáveis, tendo como oportunidade de ação o período gestacional^(24,26,27)^.

Pautando-se nessas evidências e reconhecendo a necessidade de estimular um estilo de vida saudável para conduzir os indivíduos em direção à Saúde Cardiovascular Ideal, o ACC e a AHA publicaram, em 2019, uma diretriz sobre a prevenção primária de doenças cardiovasculares baseada no controle dos fatores de estilo de vida que afetam o risco cardiovascular e outros fatores. Na publicação, a ênfase passou para os determinantes sociais da saúde no tocante a um forte foco na otimização do estilo de vida para minimizar eventos futuros de doenças cardiovasculares ateroscleróticas^(6)^.

No modelo de regressão logística, confirmou-se a associação do IMC (p = 0,02 / OR: 1,20) e da PA ideal < 120/80 (p < 0,01 / OR: 13,59) como variáveis independentes para o desenvolvimento de PE. Recentemente, tem sido proposto pelas sociedades americanas na área de gineco-obstetrícia um novo ponto de corte da PA (130/80mmHg) para o diagnóstico de PE, incorporando, assim, recomendações da AHA para detecção precoce de PE^(28)^.

Nesse sentido, visando alcançar métricas compatíveis com a Saúde Cardiovascular Ideal, reforça-se a necessidade de avaliação dos fatores de risco pré-gestacionais e pós-gestacionais para DCV, principalmente de mulheres que tenham tido gestações complicadas por PE. Também é fundamental o aconselhamento em sessões de educação em saúde promovidas pelos prestadores de cuidados pré-natais, recomendando modificações no estilo de vida e avaliações cardiovasculares regulares no período pós-parto, conforme orientações da literatura.

### Limitações do estudo

A principal limitação do estudo foi a falta de acesso em algumas maternidades para coleta dos desfechos, uma vez que muitas mulheres pariram em maternidades fora do município de Fortaleza - CE ou na rede privada, nas quais não havia autorização para coleta de dados, repercutindo na amostra final do estudo. Outra limitação importante foi a perda na realização dos exames de colesterol que não integram a rotina de pré-natal, motivo pelo qual muitas gestantes não o fizeram juntamente com a rotina nem posteriormente.

### Contribuições para a área da Enfermagem, saúde ou política pública

Diante do exposto, sugere-se a implementação de pesquisas que endossem os dados descritos e contribuam com maior evidência aos prestadores de cuidados pré natais, sobretudo o profissional enfermeiro, um dos principais atores dessa assistência na prática da atenção básica.

Quanto às contribuições para a saúde e política pública, recomenda-se conhecer a distribuição dos casos de pré-eclâmpsia, as variáveis envolvidas com esse fenômeno e a sua repercussão no *status* de saúde cardiovascular; dessa forma, pode-se orientar para a adoção de mudanças no estilo de vida. Essa prática deve acontecer desde o planejamento familiar, perpassando pelo pré-natal, até o pós-parto de mulheres que agregarem fatores de risco para PE ou que vierem a desenvolver esse agravo durante suas gestações. Com isso, objetiva-se a prevenção primária do desenvolvimento de DCV, diminuindo, assim, o risco cardiovascular e melhorando o *status* de saúde cardiovascular dessa população.

## CONCLUSÕES

No presente estudo, considerando os hábitos de vida avaliados, apenas 9,90% das gestantes tinham *status* de saúde cardiovascular ideal, enquanto 90,10% tinham algum comportamento ou fator de risco para doença cardiovascular. Entre as variáveis avaliadas, apenas IMC alterado e PA maior que 120/80mmHg constituíram-se como fatores independentes para pré-eclâmpsia, com suposição de incremento do risco cardiovascular. Não houve relação entre o desenvolvimento de PE e o *status* de saúde cardiovascular na amostra estudada.
